# The diagnostic path, a useful visualisation tool in virtual microscopy

**DOI:** 10.1186/1746-1596-1-40

**Published:** 2006-11-08

**Authors:** Thomas Schrader, Sonja Niepage, Thomas Leuthold, Kai Saeger, Karsten Schluns, Peter Hufnagl, Klaus Kayser, Manfred Dietel

**Affiliations:** 1 Institute of Pathology, University Hospital Charité, Berlin, Germany

## Abstract

**Background:**

The Virtual Microscopy based on completely digitalised histological slide. Concerning this digitalisation many new features in mircoscopy can be processed by the computer. New applications are possible or old, well known techniques of image analyses can be adapted for routine use.

**Aims:**

A so called diagnostic path observes in the way of a professional sees through a histological virtual slide combined with the text information of the dictation process. This feature can be used for image retrieval, quality assurance or for educational purpose.

**Materials and methods:**

The diagnostic path implements a metadata structure of image information. It stores and processes the different images seen by a pathologist during his "slide viewing" and the obtained image sequence ("observation path"). Contemporary, the structural details of the pathology reports were analysed. The results were transferred into an XML structure. Based on this structure, a report editor and a search function were implemented. The report editor compiles the "diagnostic path", which is the connection from the image viewing sequence ("observation path") and the oral report sequence of the findings ("dictation path"). The time set ups of speech and image viewing serve for the link between the two sequences. The search tool uses the obtained diagnostic path. It allows the user to search for particular histological hallmarks in pathology reports and in the corresponding images.

**Results:**

The new algorithm was tested on 50 pathology reports and 74 attached histological images. The creation of a new individual diagnostic path is automatically performed during the routine diagnostic process. The test prototype experienced an insignificant prolongation of the diagnosis procedure (oral case description and stated diagnosis by the pathologist) and a fast and reliable retrieval, especially useful for continuous education and quality control of case description and diagnostic work.

**Discussion:**

The Digital Virtual Microscope has been designed to handle 1000 images per day in the daily routine work of a pathology institution. It implies the necessity of an automatic mechanism of image meta dating. The non – deterministic correlation between the oral statements (case report) and image information content guides the image meta dating. The presented software opens up new possibilities for a content oriented search in a virtual slide, and can successfully support medical education and diagnostic quality assurance.

## Background

In addition to the progress made in telepathology, several institutions invest great efforts in the development and application of the so-called virtual slide technology [2,8,9,16]. A virtual slide which is the computerized image of a complete histological slide enables important applications that cannot be obtained by use of a conventional light microscope [17]. For instance, only a small group of pathologists can contemporary view the same slide, as it is usually very difficult to mark and review a specific histological feature at the microscopic level. However, especially these applications are of great importance for panel group discussions or quality assurance purposes based upon Telepathology Consultation Services, e.g. T. Konsult Pathologie of Professional Assoziation of German Pathologists [4,7,18].

**Figure 1:**
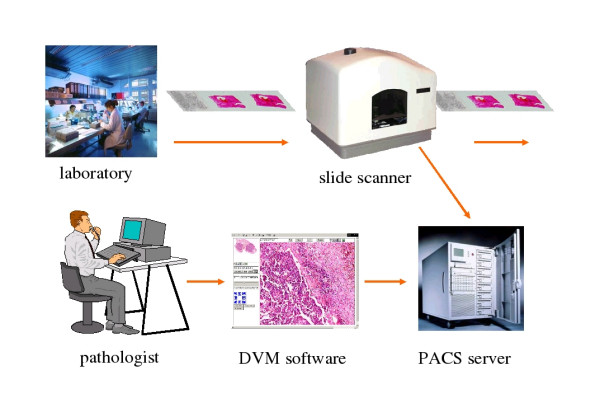
Workflow of a digitalised pathology.

**Figure 2:**
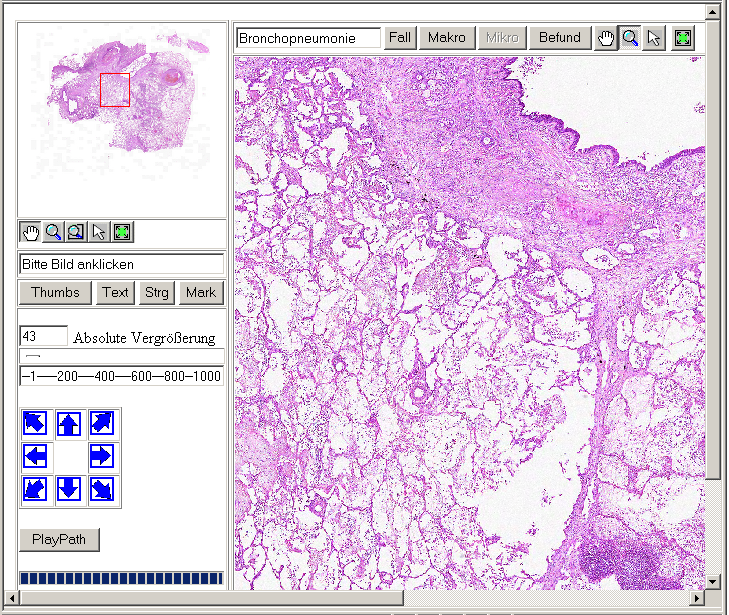
Screenshot of the Digital Virtual Microscope.

To overcome restrictions of "conventional telepatholgy" using cutaway images and microscopy the Institute of Pathology, Charité, has developed the so called "Virtual Microscope" (VM), a computer based system, that might efficiently change the paradigms in microscopy and the daily work in diagnostic pathology (figure 1) [16]. The Virtual Microscope consists of a slide scanner, an image database and a web based viewer, which allows the users to browse a complete histological slide at high resolution in real time. It permits the zoom and movement of the digital slide without any loss of image quality (figure 2) [17].

The aim of our efforts is to replace the conventional light microscopes by a digital device at the routine workplaces. These replacements will cause several implications on the workflow of the pathology institute. To start, all histological slides of a case have to be digitalized and stored in a database. The actual pathology information system has to be linked in a second step. At present this system is a highly specialized document management system only, without any functionality at the image level. The VM should narrow this gap [16,17].

The pathology reports have to be stored in a (diagnosis) database too. The reports are created when the involved pathologist observes the corresponding slide. Naturally, his report contains the description of the images. Thus, the pathology report could be considered as a metadata syntax of the digitalized slides, and the use of the pathology reports as basis for an image retrieval system is a promising logical consequence.

Normal retrieval systems use image features such as color, shape, structures and textures. Some of them deduce the content [5,15,19]. To further improve the retrieval, the implementation of a metadata structure of image information can be applied [3,6,10-13]. To achieve such improvement it is necessary to include additional information about the image content. The user of the system or creator of the image database has to feed in the necessary data. Obviously, it is easier to organizing a corresponding image database before accomplishing this stage. Extensions afterwards will often end in a scaling process. The information content of a histological image is sophisticated. Only the examining pathologist might know all significant details and can decide which metadata might be useful for further purposes.

In this paper we present a tool for an automated association of histological images, image description and the corresponding pathology report ascertained by use of the Virtual Microscope as a useful tool for image retrieval.

## Methods

The concept of the VM consists of two parts: 1) A high resolution slide scanner for scanning the histological slides and 2) a specialized viewer who transfers and processes the acquired images. The VM is written in HTML, ASP, JavaScript and VBScript. Microsoft^® ^Visual Interdev 6.0 served for the development platform. The VM user interface (client) runs within the Microsoft Internet Explorer. The database of the VM is the Microsoft SQL-Server, and the web server the Microsoft Internet Information Server.

To digitise histological slides the Zeiss Mirax Scanner with a 20× objective lens was used and a compression ratio of 20:1 is considered in order to achieving compressed images without visible loss of information. The image file type was JPEG2000. The actual loss of image information depends upon the image itself, and can be estimated to 10% at average.

Having constructed the complete virtual image, the viewing pathologist's movements within the virtual image were documented. The series of these movements were defined as "observation path", and were stored by the server of the VM. Each movement of the virtual slide was declared as individual event. The image coordinates at each event and the event time were recorded in the database.

A "normal" histopathology report includes a description of the findings and the resulting diagnosis. The description of the findings is normally dictated when viewing the slide. A digital dictation system manages the workflow of the spoken reports. The reports are usually transferred to secretaries who write and print them out. We assume in our model that the dictation process can be logged with all events (start time, end of dictation, forward and rewind), i.e. the documented time of an individual compartment of the oral description and within the observation path can link the "meaning" with the "visual appearance" of an image area. The developed software creates XML-files that include these data. In order to simulate the functionality of the diagnostic path we implemented a report editor. It creates the XML-file including the event log of the report writing, i.e. displays the oral text on the monitor.

A test-system uses a specialized editor for the creation and processing of the reports. To create the so – called dictation path, each dictated text fragment of the pathologist is stored according to the observation path. Steering commands were the time and the appending part of the report text. The reports are transferred into a XML file including the so called "dictation path" by means of this editor.

The "dictation path" contains information of actual visible slide and the recording microscopic description and diagnosis time. Several pathologists were investigated to develop the program. Some pathologists describe the case almost contemporary when viewing the slides, others view all slides of one case, dictate their findings and the corresponding diagnosis afterwards. The first procedure is mainly performed when viewing biopsy specimens, the second one when analyzing surgical specimens.

In our model we assume that the pathologist describes his findings contemporary when viewing the slides. There is a close coherence, but not deterministic, between the observation and dictation path. The combination of both paths is called the "diagnostic path".

**Figure 3:**
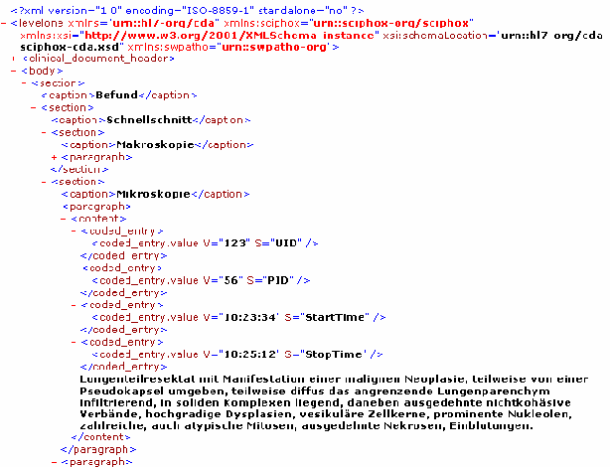
Diagnostic path in the report XML file.

Thus, the diagnostic path is the report text presented in an XML file that includes additional information about the actual viewed slide areas and each start – stop time during the diagnosis assessment (figure 3).

## Results

We developed a report-editor for automated compilation of the diagnostic path, and a retrieval tool to search for pathology reports and corresponding images, especially histological hallmarks.

**Figure 4:**
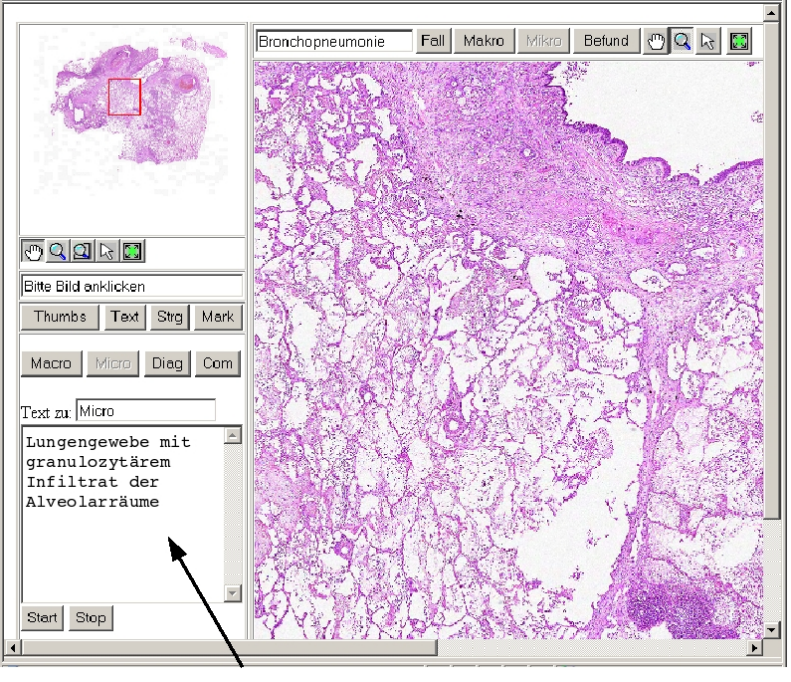
Screenshot of the Digital Virtual Microscope with the small editor area and a short part of microscopic description (arrow).

The editor was integrated in the interface of the VM. For example, the pathologist can select between four text areas according the parts of the pathology report (figure 4). The search tool consists of two additional windows: one is used to enter the search term and the second one to display the obtained results The search term will be mapped to the database of the VM. If a match is found the result window is opened and presents the list of associated image tiles as linked to the corresponding report text. Two different microscopic areas can be viewed contemporary. The search terms can be logically linked by AND or OR operations. A wildcard character (*) can be used for a more unspecific search.

Due to the close correlation between the image identifier (UDI), the start and stop times of the dictation path, and the event times of the observation path in the diagnostic path, the VM can present the searched virtual slide in the most appropriate magnification.

## Discussion

Different image archiving systems have been developed with the aim to realize a content-referred retrieval [1,3,15]. Normally these systems are based on the automated image analysis for shapes, textures, colours and structures. More sophisticated systems use techniques of case-based reasoning [10] or a specific description tool [11,12]. Each content-related metadata has to be entered by the user separately in addition to the routine diagnostic process. This performance induces a scaling problem. Theoretically and practically it is impossible to later-on add information about the images content without reviewing the whole image set, especially if more than 1000 images/day are entered and assessed as the VM is calculated to serve for. This implies the necessity of an automated or natural mechanism of meta dating the images. During the diagnostic process the pathologist has to view each slide and to comment the histological findings. Obviously, the relationship between viewing a case dictation its report, in a case which comprised one or more slides is a correlation and not deterministic. This fact is important for our model.

According to our observations two different types of correlation between viewing and diagnosing a histological slide exist: The closest correlation occurs if the pathologist dictates his findings when viewing the images. In specific situations, such as cases with multiple resected lymph nodes or cases with several stair cuts, usually all slide will not described. The pathologist only gives a statement that summarises the information of these images. The result is a weak correlation between the information content of an individual image and the image description or diagnosis.

To our knowledge, our system is the first one that automatically correlates the digital pathology report with virtual slides. Beside image parameters such as colour, texture, structure, shapes a real meta information about image content can be extracted. This metadata can be used for a more sophisticated image retrieval, for instance to compare images of a specific case with those of other cases or with reference images.

In addition to the described image retrieval the ascertained correlation of dictation and observation path can be used for a quality assurance purposes. A supervisor checks the diagnostic path and can estimate which observations were incorrect or false, and how the diagnostic process can be improved. Obviously, the diagnostic path also enhances the velocity and accuracy of the supervisor in finding and controlling histological hallmarks.

Finally, the XML-structure of the pathology report includes a distinguished basic material for linguistic analyses [14], that might be an additional interesting approach for future investigations.
